# Cloning approach and functional analysis of anti-intimin single-chain variable fragment (scFv)

**DOI:** 10.1186/1756-0500-4-30

**Published:** 2011-02-02

**Authors:** Márcio A Menezes, Karina A Aires, Christiane Y Ozaki, Renato M Ruiz, Milton CA Pereira, Patrícia AE Abreu, Waldir P Elias, Oscar HP Ramos, Roxane MF Piazza

**Affiliations:** 1 Laboratório de Bacteriologia, Instituto Butantan, Avenida Vital Brazil, 1500, São Paulo, SP, 05503-900, Brazil; 2 CEA, iBiTecs, SIMOPRO, Gif sur Yvette, France

## Abstract

**Background:**

Intimin is an important virulence factor involved in the pathogenesis of enteropathogenic *Escherichia coli *(EPEC) and enterohemorrhagic *Escherichia coli *(EHEC). Both pathogens are still important causes of diarrhea in children and adults in many developing and industrialized countries. Considering the fact that antibodies are important tools in the detection of various pathogens, an anti-intimin IgG2b monoclonal antibody was previously raised in immunized mice with the conserved sequence of the intimin molecule (int_388-667_). In immunoblotting assays, this monoclonal antibody showed excellent specificity. Despite good performance, the monoclonal antibody failed to detect some EPEC and EHEC isolates harboring variant amino acids within the 338-667 regions of intimin molecules. Consequently, motivated by its use for diagnosis purposes, in this study we aimed to the cloning and expression of the single-chain variable fragment from this monoclonal antibody (scFv).

**Findings:**

Anti-intimin hybridoma mRNA was extracted and reversely transcripted to cDNA, and the light and heavy chains of the variable fragment of the antibody were amplified using commercial primers. The amplified chains were cloned into *pGEM-T Easy *vector. Specific primers were designed and used in an amplification and chain linkage strategy, obtaining the scFv, which in turn was cloned into pAE vector. *E. coli *BL21(DE3)pLys strain was transformed with pAE scFv-intimin plasmid and subjected to induction of protein expression. Anti-intimin scFv, expressed as inclusion bodies (insoluble fraction), was denatured, purified and submitted to refolding. The protein yield was 1 mg protein per 100 mL of bacterial culture. To test the functionality of the scFv, ELISA and immunofluorescence assays were performed, showing that 275 ng of scFv reacted with 2 mg of purified intimin, resulting in an absorbance of 0.75 at 492 nm. The immunofluorescence assay showed a strong reactivity with EPEC E2348/69.

**Conclusion:**

This study demonstrated that the recombinant anti-intimin antibody obtained is able to recognize the conserved region of intimin (Int_388-667_) in purified form and the EPEC isolate.

## Background

Intimin, a 94-kDa outer membrane protein, mediates the adhesion of enteropathogenic *Escherichia coli *(EPEC) and enterohemorrhagic *Escherichia coli *(EHEC) to enterocytes. Both enteropathogens are important causative agents of diarrhea. Besides, EHEC can cause acute gastroenteritis and hemorrhagic colitis [1], and produce severe/fatal renal and neurological complications as a result of the translocation of Shiga toxins (Stx1 and Stx2) across the intestinal wall.

Intimin is encoded by the *E. coli *attaching and effacing (*eae*) gene, which is required for intimate adhesion to epithelial cells and cytoskeletal reorganization [2]. The variable 280-amino acid C-terminal sequence of intimin (Int280) defines many different intimin subtypes [3-5], and up to now, several types and subtypes of intimin have been described and designated by Greek letters [4,6-17]. In contrast, the N-terminal region of the intimin molecule is conserved and, therefore, has been used as a target for diagnostic purposes [4,18-20].

Monoclonal antibodies have been used as tools for the detection of different pathogen antigens due to their homogeneity and their unlimited production [21]. Anti-intimin IgG2b monoclonal antibody was raised in immunized mice with purified conserved intimin (int_388-667_). In immunoblotting assays, it showed excellent specificity and reacted with several serotypes of EPEC isolates. Despite good performance, the monoclonal antibody failed to detect some EPEC and EHEC isolates expressing different intimin subtypes, especially the gamma subtype [20]. In addition, monoclonal production from hybridoma is expensive and requires cell culture facilities.

Recombinant antibody (rAb) technologies involving the handling of key antibody domains constitute an option and have been increasingly used as alternatives to monoclonal antibodies (mAbs) in medical diagnostic and therapeutic applications [22]. A variety of rAb formats have been modified for specific applications, including engineered modifications to antigen binding, valency, and molecular weight (MW). One of the most popular types of rAbs is single-chain variable fragment (scFv), as it has been successfully modified into a number of different Ab formats and is easily expressed by several expression systems.

Several different molecular display formats have also been described, including phage-display [23], ribosome display [24,25] and cell-surface display [26], by which antigen-reactive Abs can be selected and affinity matured. Usually, *E. coli *is the bacterial production system of choice for small nonglycosylated rAb fragments, including scFv [27].

Regarding diarrheagenic *E. coli*, recombinant antibodies were developed against different virulence factors, which were developed for different purposes. Kühne et al. [28] produced recombinant antibodies that recognize EspA and intimin of EHEC O157:H7. These antibodies were converted to scFv format and cloned into pET22b vector. By immunoblotting, the anti-intimin scFv produced revealed the exclusive recognition of intimin gamma. The anti-EspA scFv produced relatively weak signals in immunoblotting against EspA in whole-cell preparations from serotypes O157 and O111, and no signals were produced with O127 or O86 [28].

For the treatment of bovine colibacillosis caused by enterotoxigenic *E. coli *(ETEC), Bhaskaran et al. [29] developed a recombinant anti-F5 scFv fragment that inhibits the hemmaglutination of horse red blood cells by F5 protein, which would be expected to inhibit the binding of F5-expressing ETEC to intestinal cells. According to these authors, it would provide an effective, less expensive and animal-friendly alternative as a prophylactic agent against colibacillosis. Also for ETEC, but concerning anti-LT monoclonal antibodies, Chung et al. [30] selected scFvs that specifically reacted with intermediate forms of the B subunit of LT toxin, with the potential to be used to study pentamer assembly in vivo. Thus, the generation of scFvs with differential binding specificities opens up key areas for the development of new therapeutics that can block toxin formation *in situ *and thus be used for the treatment of diarrheal diseases caused by *E. coli *and *Vibrio cholerae *[30].

Prompted by the potential applications of rAb, we studied the cloning and expression and structural and functional analysis of the recombinant form (scFv) of monoclonal anti-intimin IgG2b antibody, previously obtained from animal immunization employing conserved intimin.

## Methods

### Microorganisms and plasmids

*E. coli *JM 109 [31] was used for cloning techniques and as negative control for intimin expression. *E. coli *BL21(DE3)pLys or *E. coli *C43(DE3) were used to express the recombinant protein. EPEC E2348/69 was used as intimin expression control. *pGEM-T Easy *vector for cloning was acquired from Promega (Madison, WI, USA). The pAE expression vector was used for expression of recombinant proteins fused with six histidine residues for nickel affinity chromatography [32].

### Cloning of single-chain variable fragment (scFv) of monoclonal anti-intimin antibody

Protocols for DNA manipulation were used as described in [31]. Briefly, mRNA was extracted (Ilustra Quickprep mRNA Purification Kit - GE Healthcare, UK) and reversely transcripted to cDNA (First Strand cDNA Synthesis Kit - GE Healthcare, UK) from anti-intimin hybridoma cells [20] cultivated in RPMI medium (Invitrogen, Brazil) plus 10% fetal bovine serum in culture flasks (TPP, Switzerland) at 37°C in 5% CO_2_. Commercially available primers [Light primer mix, Heavy primer1, Heavy primer 2 (GE Healthcare, UK)] were employed to amplify the variable domain of heavy (VH) and light (VL) chains of the antibody following the manufacturer's instructions. The cDNA inserts corresponding to VL and VH were cloned into the *pGEM-T Easy *vector and sequenced using M13 forward primer (5'-GTAAAACGACGCCCAG-3') and reverse primer (5'-CAGGAAACAGCTATGAC-3'). One set of primers was designed to amplify the heavy chain variable domain, where the forward primer was complementary to the 5' terminal region of the chain and the reverse primer was complementary to the 5' terminal region of the linker (DNA fragment that encodes the [Gly_4_Ser]_3 _flexible linker that joins the heavy and light chain variable domains). Also, another set of primers was designed to amplify the light chain variable domain, where the forward primer was complementary to the 3' terminal sequence of the linker and the reverse complementary to the 3' terminal sequence of chain.

**Figure 1:**
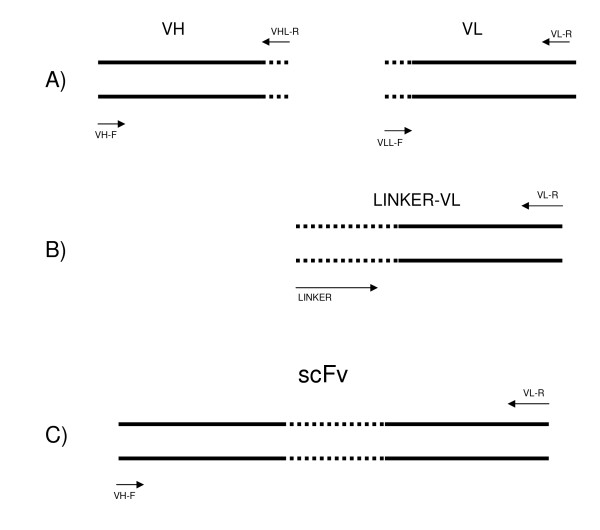
**Scheme of the PCR for scFv assembly**. **A**: Amplification of VH and VL chains using primers (arrows) containing a region complementary to the linker (dotted line). **B**: Amplification of VL chain connecting to the linker, generating the LINKER-VL fragment. **C**: Amplification and linkage of VH to the LINKER-VL and anti-intimin scFv amplification.

The light chain was amplified with the linker sequence, and then, the heavy chain was amplified with the light chain-linker sequence to generate the scFv, using the primers VH-forward and VL-reverse with the respective restriction sites (Table 1; Figure 1). Platinum *Pfx *DNA polymerase (Invitrogen, USA) was used in all PCR amplification reactions to obtain scFv. The resulting 3' non-adenylated PCR product was purified using the Ilustra GFX PCR DNA and Gel Band Purification Kit (GE Healthcare, UK) as described by the manufacturer and subjected to fill-in reaction of the 3' end with dATP. Thirty microliters of DNA were used with the addition of 1 mM dATP, 1 μL MgCl_2_, 1 μL of Taq DNA polymerase (Invitrogen, USA), 4 μL of 10× buffer for a 40-μL reaction mixture. The mixture was incubated at 72°C for 20 min. After 3' adenine anchoring, the DNA was cloned into *pGEM-T Easy *and used to transform *E. coli *JM109 competent cells. The selected colonies were subjected to PCR to confirm the presence of the scFv insert. Purified plasmids were digested using BamHI and HindIII and the scFv fragment cloned into pAE expression vector digested with the same enzymes, for expression with an N-terminal 6×His-Tag. pAE/scFv vector was used for transformation of *E. coli *BL21(DE3)pLys competent cells.

**Table 1:** Primers designed for scFv cloning and annealing temperatures.

Primers	Sequence	Annealing T°
VH Forward	5' **GGATCC**GTGCAGCTGCAGGAGTCTGG 3'	60°C
VH Reverse Linker	5' ACCGCCTCCACCGGAGACGGTGACCGTG 3'	76°C
VL Forward Linker	5' GGTGGCGGATCGGACATTGTGCTGACC 3'	78°C
VL Reverse	5' *AAGCTT****TTA***GTTTGATTTCCAGCTTGGTGCC 3'	70°C

Heavy chain forward primer contains the restriction site BamHI (bold). At the light chain reverse primer was inserted the restriction site HindIII (italic) and a stop codon (bold/italic)

### Recombinant antibody (anti-intimin scFv) expression, solubility and immunodetection

pAE-scFv was introduced into *E. coli *BL21(DE3)pLys. The clone was cultivated (37°C, 300 rpm) in 400 ml of 2YT broth containing 100 μg/ml ampicillin and 20 μg/ml chloramphenicol until OD_600 _= 0.5. At this point, IPTG was added to a final concentration of 1 mM, and the culture was continued for an additional 5 h under the same conditions. The culture was centrifuged (4,500 × g, 15 min, 4°C), the pellet was resuspended in 30 ml of PBS, pH 7.4, and the bacterial cells were disrupted in a French press under 2,000 psi (Thermo Scientific, USA). The bacterial extract was centrifuged (12,000 × g, 30 min, 4°C), and the pellet was resuspended in 30 ml solubilizing buffer (50 mM Tris-HCl, pH 8.0; 200 mM NaCl; 5 mM imidazole; and 8 M urea) for 18 h at 4°C with constant mixing (100 rpm). The bacterial extract before and after induction, as well as soluble and insoluble protein fractions, was analyzed by 12% SDS/PAGE followed by Coomassie Blue staining (Bio-Rad, USA) or transferred to nitrocellulose membrane for Western blot assay (Bio-Rad, USA). The membrane containing the bacterial protein extracts was blocked with blocking buffer (5% skim milk diluted in PBS) at 37°C for 30 min and incubated with 1:3000 anti-histidine mouse antibody (GE Healthcare, UK) diluted in blocking buffer at 37°C for 30 min. The membrane was washed with PBS plus 0.05% Tween 20 (PBST) 3 times for 5 min, followed by 1:2000 goat anti-mouse IgG conjugated to peroxidase (Zymed-Invitrogen, USA) diluted in blocking buffer at 37°C for 30 min. The membrane was then washed and revealed with diaminobenzidine (DAB) plus H_2_O_2_.

### Purification and refolding of the recombinant scFv anti-intimin antibody obtained

The solubilized recombinant scFv was centrifuged (12,000 × g, 4°C, 30 min) and the supernatant was purified by immobilized metal affinity chromatography (IMAC) using Ni-Sepharose resin (GE Healthcare, UK). The column was washed with solubilization buffer to eliminate nonspecific proteins. Next, the specific protein was eluted with elution buffer (50 mM Tris-HCl, pH 8.0; 200 mM NaCl; 500 mM imidazole; and 8M urea). Purified recombinant scFv antibody was analyzed by 12% SDS/PAGE, as described above.

The purified scFv was dialyzed against solubilization solution containing 5 M urea for 12 h at room temperature using dialysis membrane with 8,000 Da cut-off. Step-wise dialysis was conducted by changing buffers with decreasing urea concentrations (3 M, 2 M, 1 M, 0.5 M and no urea). Considering the 1 M urea step, oxidized glutathione (375 μM) was added to the buffer to facilitate stable disulfide bond formation. The solubilized scFv was concentrated in polyethylene glycol 6000 (PEG 6000) and quantified by the Warburg-Christian method [33].

### Tertiary and quaternary structure predictions

The tertiary structures of the conserved intimin N-terminal region (int_388-667_) were predicted, based on amino acid sequence deduced from published DNA sequence [GenBank:CAS11487] and anti-intimin scFv generated in the present work [GenBank:GU722326] (Table 2) using I-TASSER server http://zhang.bioinformatics.ku.edu/I-TASSER. The quaternary structure of the complex was calculated using ZDOCK 3.0 full search [34]; http://zlab.bu.edu/zdock, following a local refinement protocol of the best evaluated model implemented in Rosetta Dock [35]; http://graylab.jhu.edu/docking/rosetta.

**Table 2:** VH, linker and VL nucleotide sequence and predicted amino acid sequence of VH, linker, VL and conserved intimin (Gly388 - Lys667)

Nucleotide Sequences	
CHAINS	SEQUENCES [GenBank:GU722326]

VH	GTGCAGCTGCAGGAGTCTGGACCTGAGGTGGTGAAGCCTGGGACTTCAGTGAAGATATCCTGTAAGGCTTCTGGATACACGGTCACTGACTACTACATGAACTGGGTGAAGCAGAGCCATGGAAAGAGCCTTGAGTGGATTGGAGATATTAATCTTGACAATCGTGATTGTAGTTATAACCAGAAGTTCCAGGACAAGGCCACATTGACTGTAGACAAGTCGTCCAGCACAGTCTACATGGAGATCCGCAGCCTGACTTCTGAGGACTCTGCAGTCTATTACTGTGCAAGTCAACTGGGTCACTGGGGCCAAGGGACCACGGTCACCGTCTCC
Linker	GGTGGAGGCGGTTCAGGCGGAGGTGGCTCTGGCGGTGGCGGATCG
VL	GACATTGTGCTGACCCAGTCTCCAGCTTCCTTAGCTGTATCTCTGGGGCAGAGGGCCACCATCTCATACAGGGCCAGCAAAAGTGTCAGTACATCTGGCTATAGTTATATGCACTGGAACCAACAGAAACCAGGACAGCCACCCAGACTCCTCGTCTATCTTGTATCCAACCTAGAATCTGGGGTCCCTGCCAGGTTCAGTGGCAGTGGGTCTGGGACAGACTTCACCCTCAACATCCATCCTGTGGAGGAGGAGGATGCTGCAACCTATTACTGTCAGCACATTAGGGAGCTTACACGTTCGGAGGGGGCACCAAGCTGGAAATCAAAC

**Amino Acid Sequences**	

CHAINS	SEQUENCES

VH	VQLQESGPEVVKPGTSVKISCKASGYTVTDYYMNWVKQSHGKSLEWIGDINLDNRDCSYNQKFQDKATLTVDKSSSTVYMEIRSLTSEDSAVYYCASQLGHWGQGTTVTVS
Linker	GGGGSGGGGSGGGGS
VL	DIVLTQSPASLAVSLGQRATISYRASKSVSTSGYSYMHWNQQKPGQPPRLLVYLVSNLESGVPARFSGSGSGTDFTLNIHPVEEEDAATYYCQHIRELTRSEGAPSWKSN
Int_388-667 _[GenBank:CAS11487]	GIDYRHGTGNENDLLYSMQFRYQFDKPWSQQIEPQYVNELRTLSGSRYDLVQRNNNIILEYKKQDILSLNIPHDINGTERSTQKIQLIVKSKYGLDRIVWDDSALRSQGGQIQHSGSQSAQDYQAILPAYVQGGSNVYKVTARAYDRNGNSSNNVLLTITVLSNGQVVDQVGVTDFTADKTSAKADGTEAITYTATVKKNGVAQANVPVSFNIVSGTAVLSANSANTNGSGKATVTLKSDKPGQVVVSAKTAEMTSALNANAVIFVDQTKASITEIKADK

### Functionality assays

The functionality of the purified anti-intimin scFv was assayed by ELISA and immunofluorescence.

#### Sandwich ELISA assay with polyclonal anti-intimin antibody

A polystyrene plate MaxiSorp (Nunc, Denmark) was coated with 0.05 M carbonate/bicarbonate buffer, pH 9.6, containing serial dilutions of recombinant anti-intimin scFv antibody 1:200 (1 μg) to 1:1600 (125 ng) for 18 h at 4°C. The wells were blocked with 5% skim milk diluted in PBS buffer and then, 20 μg/ml purified intimin (Int_388-667_) was added and incubated for 2 h at 37°C. The polyclonal rabbit anti-intimin IgG enriched fraction was added in serial dilutions from 1:200 (7.5 μg) to 1:3200 (470 ng) and incubated for 30 min at 37°C. The detection was done with goat anti-rabbit IgG conjugated to peroxidase (Invitrogen, USA) diluted 1:5000 and revealed with ο-phenylenediamine (OPD). The absorbance was measured at 492 nm and the reaction without purified intimin was taken for background noise subtraction. Between each reaction step, washing was done with PBS buffer containing 0.05% Tween 20. The purified intimin used in the assays was obtained as described by Menezes et al [20].

#### Immunofluorescence

EPEC E2348/69 was cultivated in Luria-Bertani broth (LB) at 37°C for 18 h with constant shaking (250 rpm). One milliliter of the growth culture was centrifuged at 12,000 × g for 10 min and washed three times in PBS buffer. The pellet was permeabilized in 4% Triton X-100 under constant shaking (200 rpm) for 10 min and washed twice in PBS buffer, followed by 1% ρ-formaldehyde incubation for 20 min followed by two washes with PBS. Immunofluorescence reaction was performed using 44 μg/ml recombinant anti-intimin scFv for 1 h at room temperature, followed by anti-histidine mouse antibody incubation at 1:2000 dilutions for 1 h at room temperature. Goat anti-mouse IgG conjugated to fluorescein isothiocyanate (FITC) (Sigma, USA) at 1:100 dilution was added and the mixture incubated for 1 h at room temperature. One drop of the bacterial suspension was placed on a glass slide and examined with a fluorescence microscope (Axioskop-Zeiss, Germany) under 1000× magnification. As negative control, the assay was performed with *E. coli *K12 JM109.

## Results

### Cloning strategy of the recombinant anti-intimin scFv

The amplified fragments corresponding to VL and VH were cloned into *pGEM-T Easy *vector and sequenced using the M13 primers (forward and reverse). The predicted amino acid sequences of VL and VH (Table 2) were analyzed in the NCBI database (blast.ncbi.nlm.nih.gov) using the BLAST tool, and a homology of over 90% was verified with the published data of the variable region of antibodies of the mice species *Mus musculus*. Conserved regions of heavy and light chain variable domains were used to design new primers containing appropriate restriction sites for subsequent cloning into expression vectors (Table 1).

Amplification products of approximately 300 bp were obtained using these specific primers, corresponding to the variable domains of light and heavy chains (VL and VH). In order to obtain and connect the light and heavy chains variable domains by the linker sequence, four PCR reactions were performed, resulting in a product of approximately 700 bp (Figure 1), corresponding to the anti-intimin scFv (scFv-int), which was subsequently cloned into pAE expression vector. Transformed clones were screened by colony PCR and sequenced (data not shown).

### Expression of the recombinant anti-intimin scFv

**Figure 2:**
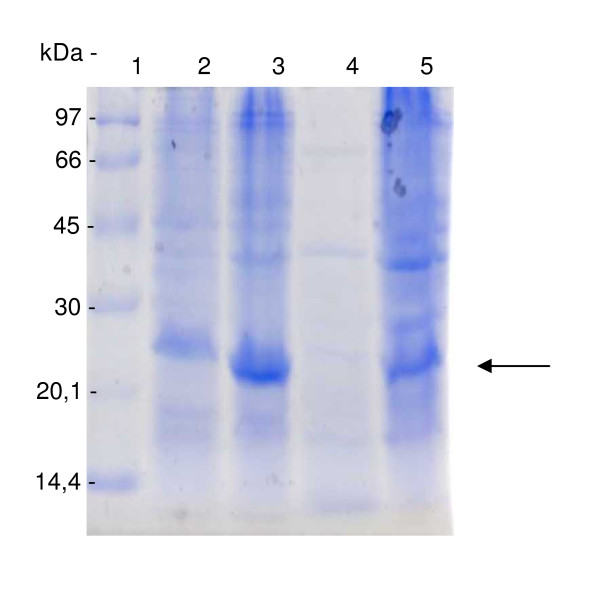
**Expression analysis of recombinant scFv-intimin**. *E. coli *BL21(DE3)pLysS total extracts analyzed by electrophoretic profile in 12% SDS/PAGE. **Lane 1**. Molecular weight marker (LMW 97 to 14.4 kDa). **Lane 2**. Total bacterial extract before induction. **Lane 3**. Total bacterial extract 5 h after induction. **Lane 4**. Soluble fraction of bacterial extract. **Lane 5**. Insoluble fraction of bacterial extract (inclusion bodies) treated with 8 M urea. Recombinant protein of interest indicated by the arrow.

**Figure 3:**
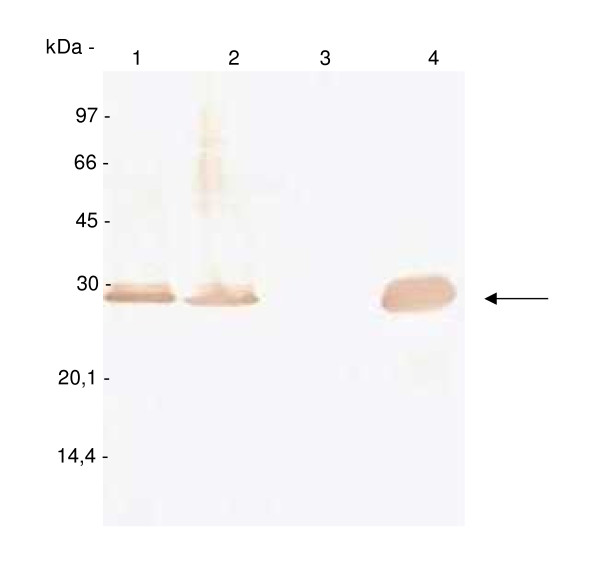
**Immunodetection of recombinant scFv-intimin**. *E. coli *BL21(DE3)pLysS extracts were submitted to 12% SDS/PAGE, transferred to nitrocellulose membrane and detected by immunoblotting with mouse anti-His antibody. **Lane 1**. Total extract as control of pre-induction. **Lane 2**. Total extract of bacterial cells after induction. **Lane 3**. Soluble fraction of bacterial extract. **Lane 4**. Insoluble fraction of bacterial extract (inclusion bodies) treated with 8 M urea. Recombinant protein containing 6-histidine residues detected by anti-His antibody is indicated by the arrow.

To confirm the presence of the recombinant scFv fused to 6 histidine residues in the extract of *E. coli *BL21(DE3)pLys, samples were analyzed by 12% SDS/PAGE (Figure 2) and immunodetected using mouse anti-His antibody (Figure 3). The results showed the expression of a 26.5 kDa protein, the predicted size of anti-intimin scFv as inclusion bodies in the post-induction fraction (Figure 2, **lanes 3 and 5 **and Figure 3**, lanes 2 and 4**).

### Purification of the recombinant anti-intimin scFv

**Figure 4:**
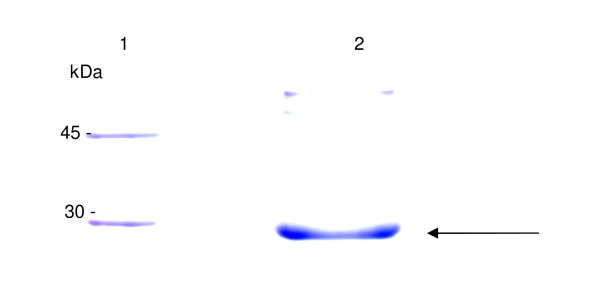
**SDS/PAGE analysis of purified recombinant scFv-intimin after purification by affinity chromatography to nickel-containing Sepharose**. **Lane 1**. Molecular weight marker (LMW 97 to 14.4 kDa). **Lane 2**. Fraction of purified anti-intimin scFv, with 26.5 kDa indicated by the arrow.

The insoluble protein produced was solubilized using 8 M urea buffer and purified by immobilized metal affinity chromatography (IMAC) using Ni-Sepharose resin. The purified protein was then refolded, quantified and subjected to analysis by SDS/PAGE (12%), revealing a protein of 26.5 kDa, predicted for scFv (Figure 3, **lane 4 **and Figure 4**, lane 2**). The final process yield was approximately 10 mg of refolded scFv per liter of bacterial culture.

### Structural and functional analysis of the recombinant anti-intimin scFv

**Figure 5:**
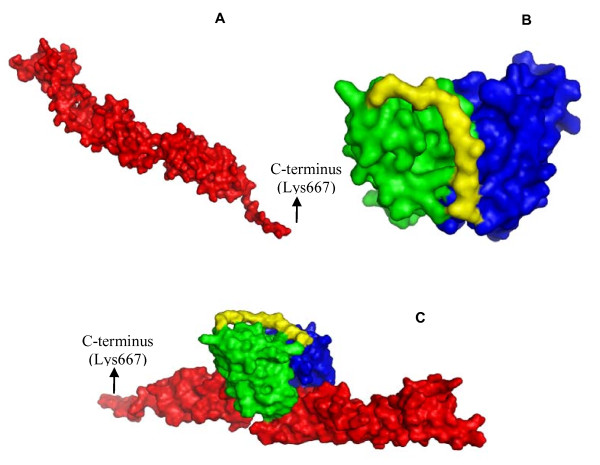
**scFv-intimin and intimin molecular structure**. Constructed models based on the predicted structures of conserved intimin and recombinant anti-intimin scFv. **A**. Conserved intimin (int_388-667_) [GenBank:CAS11487]. **B**. Anti-intimin scFv (heavy chain in green, light chain in blue and linker in yellow). **C**. Interaction between conserved intimin and anti-intimin scFv as predicted by the programs ZDOCK and Rosetta Dock.

The overall *in silico *analysis of the constructed models revealed expected features corresponding to scFv (Figure 5A) and intimin domain arrangement that is typically found in homologous molecules (Figure 5B). Both models were subjected to calculations concerning complex formation. The final complex structure suggested that CDR-H3, CDR-L1 and CDR-L2 (the last to a lesser extent) would be mainly involved in the recognition of a region corresponding to the connection between two intimin_388-667 _Ig-like domains (Figure 5C).

**Figure 6:**
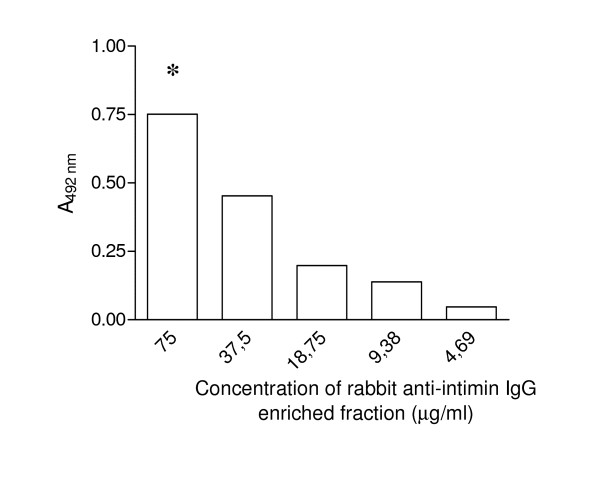
**Functional assay of anti-intimin scFv by indirect sandwich ELISA**. Microplates were coated with a serial dilution of anti-intimin scFv, followed by incubation with 2 μg/mL of purified intimin and a serial dilution of rabbit anti-intimin IgG enriched fraction antibody. The graph shows the best fit titration curve of 275 ng of anti-intimin scFv reacting with 2 μg of purified intimin in which the optimal absorbance was defined by 7.5 μg rabbit anti-intimin IgG enriched fraction antibody (*).

**Figure 7:**
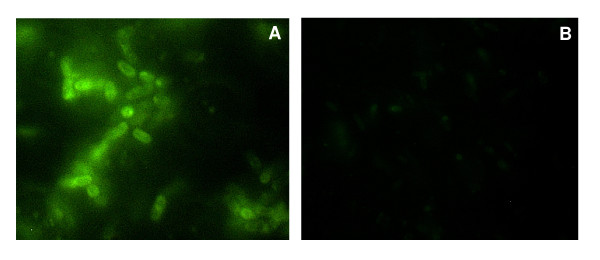
**Functional assay of scFv-intimin by immunofluorescence**. **A**. EPEC E2348/69 strain permeabilized with 4% Triton X-100 followed by incubation with 4.4 μg of anti-intimin scFv. The reaction was developed using anti-His antibody and anti-mouse IgG conjugated to FITC. **B**. *E. coli *K12 JM109 (negative control) assayed as described for **A**.

The reactivity of the recombinant anti-intimin scFv was investigated by sandwich ELISA assay in a checkerboard titration. The reactivity with 2 μg of purified intimin was observed until 125 ng of scFv (data not shown). The best fit titration curve corresponded to 275 ng of scFv, in which the optimal absorbance was 0.75 using 7.5 μg of polyclonal rabbit anti-intimin IgG enriched fraction (Figure 6). The functionality of recombinant scFv was tested by an immunofluorescence assay that showed a strong reactivity of the permeabilized EPEC E2348/69 cells with the scFv antibody (Figure 7). As expected, the negative control, *E. coli *K12, was not stained using the scFv antibody. These results demonstrate that recombinant scFv produced in this work is functional and recognizes either the conserved region of intimin, used in the ELISA assay, or native intimin on the permeabilized EPEC E2348/69 cells.

## Discussion

Serotyping has been widely applied in the diagnosis of gastrointestinal diseases caused by EPEC and EHEC, but cannot be used for identifying these groups conclusively [36]. Therefore, identification of the characteristic virulence genes is an obvious choice for detection. PCR to detect one or multiple virulence genes in the same reaction has been a successful method for the detection of EPEC and EHEC, as well as other DEC pathotypes. Nevertheless, gene detection does not assure expression of the corresponding virulence factor [20,37,38]. Among the methods for detection of virulence factor expression, immunoassays can be considered the first alternative, for which polyclonal and/or monoclonal antibodies are raised [19,39-41].

The great genetic diversity of intimin, demonstrated by the description of at least 27 subtypes, makes difficult its molecular detection, which is dependent on the primers sequences [11,17]. Moreover, a negative PCR result may not indicate that intimin is absent. For this reason the conserved region of this protein (Int_388-667_) was used as antigen for the development of a potential universal anti-intimin antibody able to detect the current known intimin subtypes and further uncharacterized ones [18-20].

In a previous work, even with an affinity constant of 1.3 × 10^-8 ^M and a good detection limit with purified intimin, monoclonal anti-intimin IgG2b antibody detected 78% of a wide range of EPEC and EHEC intimin-expressing strains [20]. These promising results prompted the objective of this work, the production of a functional scFv (corresponding to monoclonal anti-intimin antibody) intended for diagnosis purposes.

To outline our strategy, the pAE vector was used for scFv expression which was constructed on the basis of the pRSET vector containing the origin of replication of high copy number, with the multiple cloning site containing 6 histidine residues from the pET3-His vector [32]. This vector was used successfully by others [32,42-48], and despite the fact that in this study the protein was expressed in an insoluble form, a good yield was obtained.

In order to analyze the expression level of recombinant scFv in *E. coli*, different host strains were used, namely BL21(DE3)pLys and C43(DE3). These strains prevent the escape of expression and contribute to an over expression of the recombinant protein after induction. The analysis of expression in the C43(DE3) strain showed the presence of a protein of predicted molecular weight of 26.5 kDa, and another of smaller size (data not shown). This could be due to action of a protease that could be expressed in this strain. Another hypothesis is that the presence of rare codons in the scFv sequence could lead to truncated protein forms in this strain. On the other hand, despite the presence of some protein before induction, recombinant *E. coli *BL21(DE3)pLys successfully expressed scFv with the expected molecular weight. These results showed that *E. coli *BL21(DE3)pLys expressed more protein and only the predicted one. Thus, this strain was chosen for the expression of scFv anti-intimin.

Expression in the reducing environment of the cytoplasm can be achieved at high concentrations but often results in the formation of inclusion bodies (*i.e*., reduced and unfolded proteins), where the product requires solubilization with denaturing agents (*e.g.*, 8 M urea) and subsequent refolding, by dialysis, of the denaturing agent in the presence of a redox pair (*e.g.*, reduced and oxidized glutathione). Refolding efficiency varies for different antibodies [49]. The anti-intimin scFv obtained in this work was also expressed as insoluble bodies, and thus, it was necessary to perform denaturing solubilization with subsequent refolding steps.

The method chosen for recovering insoluble protein was the solubilization in Tris-HCl buffer containing 8 M urea, followed by purification and step-wise dialysis for the refolding of the purified protein by decreasing urea concentration. Anti-intimin scFv was expressed with 6 histidine residues at the N-terminal, for the purpose of purification by metal affinity. In addition to purification, the His-tag is an epitope for detection using specific antibodies. To demonstrate the functionality of the scFv by ELISA, purified intimin conserved domain (Int_388-667_) is the best reagent for detection. However, the simple fact that the purified intimin also contains His-tag clearly impairs scFv detection, since the former could create false positive results.

To overcome this technical limitation, the strategy used was the sandwich indirect ELISA assay using the rabbit IgG enriched fraction or monoclonal anti-intimin. This protocol consisted of coating the plate with anti-intimin scFv, followed by incubation with purified intimin and rabbit polyclonal anti-intimin IgG, without the use of anti-His. Results showed that anti-intimin scFv is a functional antibody, and as expected, the polyclonal antibody does not compete with the scFv for the same epitope. On the other hand, no reactivity was observed when the monoclonal anti-intimin antibody was used, suggesting that this antibody competes with the scFv for the same intimin epitope (data not shown). Moreover, anti-intimin scFv was tested by immunofluorescence. This antibody recognized the EPEC E2348/69 strain but not *E. coli *K-12 after outer membrane permeabilization, showing its specificity for intimin.

The results of this study allow us to conclude that recombinant anti-intimin scFv was able to recognize the conserved region of purified intimin (Int_388-667_) and intimin in an EPEC isolate. Subsequent tests underway in our laboratory could demonstrate if the scFv reacts with the different types of intimin expressed by EPEC and EHEC. These data will be extremely important, since the recombinant scFv described so far is derived from spleen cell-cDNA from rabbits immunized with the intimin variable portion of O157:H7 and recognize only gamma intimin [28]. It is worth mentioning that our previous results already showed that monoclonal anti-intimin antibody recognizes a broad variety of EPEC and EHEC strains [20], and as the anti-intimin scFv described herein is derived from it, this rAb is a promising tool for EPEC/EHEC diagnosis.

## Conclusions

In conclusion, we successfully obtained a recombinant anti-intimin scFv with high specificity for intimin, detecting the conserved region of intimin (Int_388-667_) in the purified form and intimin on an EPEC isolate. Thus, this rAb is a very promising tool for EPEC/EHEC diagnosis and research purposes. Also, the deduced amino acid sequences of CDRs may also help us to create other scFv fragments with even higher affinity and specificity for intimin by site-directed mutagenesis.

## Abbreviations

EPEC: enteropathogenic *Escherichia coli*; EHEC: enterohemorrhagic *Escherichia coli*; ETEC: enterotoxigenic *Escherichia coli*; *eae*: *E. coli *attaching and effacing; Ab: antibody; rAb: recombinant antibody; mAb: monoclonal antibody; MW: molecular weight; scFv: single chain variable fragment; VH: heavy chain variable domain; VL: light chain variable domain; EspA: *E. coli *secreted protein A; LT: heat-labile toxin; cDNA: complementary DNA; NCBI: National Center for Biotechnology Information; BLAST: Basic Local Alignment Search Tool; PCR: polymerase chain reaction; IMAC: immobilized metal affinity chromatography; SDS: sodium dodecyl sulfate; PAGE: polyacrylamide gel electrophoresis; CDR: complementarity determining region; ELISA: enzyme-linked immunosorbent assay; RPAS: recombinant phage antibody system; His: histidine; IPTG: isopropyl β-D-thiogalactopyranoside; RPMI: Roswell Park Memorial Institute; dATP: deoxiadenosine triphosphate; PBS: phosphate-buffered saline; PBST: PBS plus Tween 20; DAB: diaminobenzidine; PEG: polyethylene glycol; OPD: ο-phenylenediamine; LB: Luria-Bertani broth; FITC: fluorescein isothiocyanate.

## Competing interests

The authors declare that they have no competing interests.

## Authors' contributions

MAM and KAA contributed equally to this work, performing all cloning approaches and protein expression, and contributed to the preparation of the manuscript. CYO contributed to the molecular study, performed all the ELISA assays, and contributed to the preparation of the manuscript. RMR and PAEA contributed to the molecular assays. MCAP performed all immunofluorescence assays. WPE contributed to the preparation of the manuscript and critically reviewed it. OHPR contributed to the tertiary and quaternary structure predictions and to the preparation of the manuscript. RMFP conceived and designed the study and oversaw the preparation of the manuscript. All authors read and approved the final manuscript.
